# How mothers and fathers support adult childhood cancer survivors: parental attitudes, involvement, and motivation toward long-term follow-up care (results from the Swiss Childhood Cancer Survivor Study – Parents)

**DOI:** 10.1007/s00520-025-10040-8

**Published:** 2025-10-29

**Authors:** Julia Baenziger, Anica Ilic, Tamara Diesch-Furlanetto, André O. von Bueren, Grit Sommer, Gisela Michel, Manya J. Hendriks

**Affiliations:** 1 Faculty of Health Sciences and Medicine, University of Lucerne, Lucerne, Switzerland; 2 Heart Centre for Children, The Sydney Children’s Hospital Network, Sydney, NSW Australia; 3 Departemnt of Pediatic Oncology/Hematology, Children’s Hospital of Basel, Basel, Switzerland; 4 Department of PediatricsGynecology and Obstetrics, Division of General Pediatrics, Pediatric Hematology and Oncology Unit, University Hospitals of Geneva, Geneva, Switzerland; 5 Department of PediatricsGynecology and Obstetrics, Cansearch Research Platform for Pediatric Oncology and Hematology, Faculty of Medicine, University of Geneva, Geneva, Switzerland; 6 Institute for Social and Preventive Medicine, University of Bern, Bern, Switzerland

**Keywords:** Follow-up, Parents, Childhood cancer, Cohort, Health promotion, Long-term

## Abstract

**Purpose:**

Many childhood cancer survivors (CCS) do not attend long-term follow-up (LTFU) care. We examined (1) the involvement of mothers and fathers, (2) their attitudes towards LTFU, (3) how they motivated their adult children to attend, (4) and parents’ perceptions of the healthcare professionals involved and the decision to end LTFU care.

**Methods:**

A population-based sample (Swiss Childhood Cancer Registry) of parents of long-term CCS (> 5 years post-diagnosis, ≥ 20 years at study) responded to a questionnaire. Multiple-choice and open-ended questions were analysed using descriptive statistics, chi2 comparisons, and qualitative content analyses.

**Results:**

Of 302 families, 190 fathers (40.7%) and 276 mothers participated. One in four (26.1%) parents were involved in LTFU, providing medical, preventative/practical, and emotional support (mothers > fathers, *p* = 0.013). Parents of LTFU attenders were pleased with attendance (94.3%), providing them with reassurance about their child’s health. Parents of non-attenders did not wish their child attended LTFU (74.7%), because of their perceptions (e.g., ‘being cured’), respect for the child’s decision, or the need to move on. Parents (53.5%) motivated attenders (mothers > fathers, *p* = 0.002) by talking about importance, helping to schedule, and reminding. General practitioners (64.3%) and adult oncologists (31.9%) most often provided LTFU. The decision to end LTFU was made by the treating physician (53.4%), survivors (18.4%), or shared decision-making (17.5%).

**Conclusion:**

There is unused potential for parents to motivate their children to participate in LTFU. The variety of LTFU models can be difficult to navigate; thus, working to improve visibility and encouragement might help increase attendance.

## Background

Childhood cancer is a family matter. Even years after successful treatment, parents may continue to have concerns related to cancer relapse or late effects, and remain engaged in their child’s long-term care [1]. For example, in Switzerland, among adolescent childhood cancer survivors (CCS, 11–17 years), parental involvement was still widespread (92%) [1]. Mothers in particular are often reported as accompanying their children to long-term follow-up care (LTFU) visits [2, 3]. Social norms, including parents’ support and expectations, have been associated with higher intention for LTFU attendance [4]. Given the life-long risk of medical late effects, regular LTFU is essential [5, 6]. Yet, despite recommendations, few CCS attend LTFU [6–10].

Little is known about how parental involvement continues once survivors reach adulthood, or which factors influence whether mothers and fathers remain engaged and encourage survivors’ attendance [11, 12]. The present study addresses this gap by describing parent self-reported involvement in their child’s LTFU care and type of specialists consulted.

## Methods

### Aims & study design

In this cross-sectional study, we combined closed and open-ended survey questions to investigate: (1) parental involvement in their adult child’s LTFU, (2) parental attitude towards their child’s LTFU attendance, and (3) parental behaviour and reasoning in motivating their child to attend LTFU; including potential differences among mothers and fathers of long-term CCS. Additionally, we asked parents (4) which healthcare professionals are involved in the child’s LTFU or who decided to end LTFU.

### Population

This study is part of the Swiss Childhood Cancer Survivor Study on the health and well-being of Parents of long-term CCS (SCCSS-Parents) [13]. A population-based sample of parents of long-term CCS was identified at the Swiss Childhood Cancer Registry of Switzerland (SCCR, https://www.childhoodcancerregistry.ch) [14]. Eligibility criteria included being a parent of a child (≤ 16 years old and Swiss resident at cancer diagnosis, at least five years post-diagnosis and off treatment, and ≥ 20 years old in 2016).

### Procedure

**Fig. 1:**
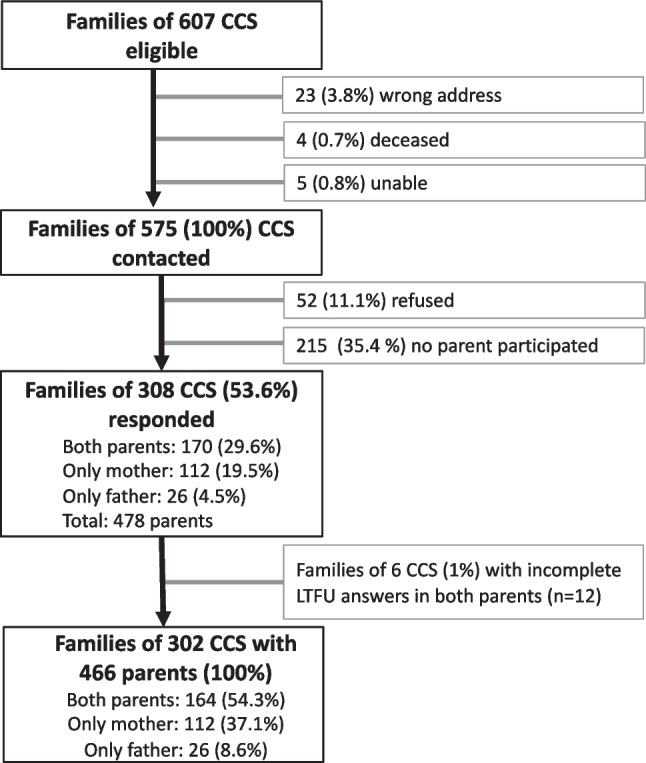
Recruitment flowchart of participating parents of childhood cancer survivors (CCS, aged > 20 years) to study long-term involvement in long-term follow-up care

Parents of 575 long-term CCS received an information letter from their former treating clinic with an invitation to participate (Fig. 1). Two weeks later, each parent was invited to complete a questionnaire individually. Non-responders received up to two reminders, sent approximately two and ten weeks later (01.2017–02.2018). Study materials were available in German, French, or Italian to cover the main national languages. Returned questionnaires were entered in EpiData, with 10% double-entred to ensure accuracy.

### Measurements

#### LTFU

Parents reported whether their child still *attended LTFU*: yes, regularly; yes, irregularly; no; or unaware. Parents who responded to questions about their child’s LTFU attendance were categorized into parents of attenders (regular/irregular) and parents of non-attenders (no/unaware). Parents who reported being unaware of their child’s LTFU status were categorized as non-attenders, because lack of awareness was considered to indicate absence of parental engagement in LTFU.

Parental *involvement* in LTFU: Parents reported whether they were involved (yes/no) and how (open question).

A*ttitude* towards LTFU attendance: Parents of attenders reported whether they are pleased (yes/no/unsure) about their child’s attendance, and parents of non-attenders, whether they would desire that their child attends LTFU (yes/no). *Reasons* for their attitudes towards LTFU were assessed in an open question.

**Fig. 2:**
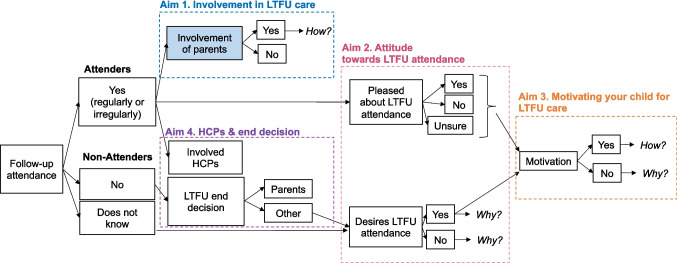
Themes used in the open-ended questions aiming to better understand parental involvement in long-term follow-up of adult childhood cancer survivors. *Abbreviations: LTFU, long-term follow-up; HCP, healthcare professional*

*Motivating*: Parents reported if they are *motivating* their child to attend LTFU (yes/no), and how (open question). Non-motivating parents were asked to describe their reasoning (Fig. 2).

*Healthcare professionals involved*: Parents of attenders were asked to indicate which healthcare professionals were involved in the child’s LTFU (multiple choice: general practitioner, paediatric oncologist, adult oncologist, gynaecologist, endocrinologist, ophthalmologist, neurologist, nephrologist, and ‘other’ to specify additional professionals). Parents of non-attenders were asked who decided to end LTFU (we [parents]/my child/paediatric oncologist/other [to be specified]).

#### Cancer-related characteristics

Cancer-related characteristics were available from the SCCR: cancer diagnosis, treatment, age at diagnosis [years], time since diagnosis [years], and relapse (yes/no). Cancer diagnoses were classified according to the ICCC-3 [15] and categorized into leukaemia, lymphoma (including Langerhans cell histiocytosis), central nervous system tumour, and solid tumour. Treatment was categorized into: surgery only, chemotherapy (may have had surgery), radiotherapy (may have had surgery and/or chemotherapy), and stem cell transplantation (may have had surgery and/or chemotherapy and/or radiotherapy). Parents indicated in the questionnaire whether their child experienced late effects (yes/no).

#### Parental characteristics

Participants reported their parental role (Mother/Father), age (years), questionnaire language (German/French/Italian), migration background (defined as not being a Swiss citizen, not a Swiss citizen since birth, or not born in Switzerland), education (compulsory schooling/vocational training/upper secondary or university degree), employment status (yes/no [includes in education and retirement]), monthly household income in Swiss francs (CHF, < 6000/≥ 6000), number of children (≤ 2/> 2), civil status (single/married/widowed or divorced), whether they were living in a partnership (yes/no), and whether they perceived themselves and their child as having a chronic health condition (yes/no) [14].

### Analysis

To qualitatively describe Aim 1–3 (parents involvement, attitude, motivating their child), open-ended responses were analysed using the principles of content analysis with ATLAS.ti 22 [16, 17] following the approach from Kuckartz [18]. First, AI and MH familiarized themselves by reading all answers to one of the questions, and developed a coding scheme together by combining deductive (derived from our research questions) and inductive codes (derived from the data). Second, preliminary codes were assigned to all questions independently, by either AI or MH, creating additional codes where necessary (inductive and deductive phases). To ensure rigor, all authors exchanged files and reviewed the identified codes, discussing them in iterative meetings, and resolving discrepancies until consensus was reached to refine the coding scheme. Third, AI and MH collaboratively finalized the coding guide. Finally, the codes were categorized into overarching themes presented in the results after a discussion with all authors, and representative quotes were selected. To ensure the accuracy of participants’ quotes, back-to-back translation was performed [19]. For aim 4 (healthcare professionals and end of LTFU) we grouped and counted similar professions. To describe the study population (Table 1) and Aim 4 (Table 3), we used descriptive statistics. We compared mothers’ and fathers’ answers using chi^2^ tests. All statistical analyses were performed using Stata 16.0

**Table 1:** Socio-demographic characteristics of parents of long-term childhood cancer survivors

Parents of Childhood Cancer Survivors (*N* = 466)		
	*n*	%
Gender		
Mother	276	59.2
Father	190	40.8
Age category, years		
36–55	71	15.2
56–65	238	51.1
66 +	153	32.8
Unknown	4	0.9
Language		
German	344	73.8
French	105	22.5
Italian	17	3.7
Migration background		
No	384	82.4
Yes	56	12.0
Unknown	26	5.6
Civil Status		
Single	5	1.1
Married	369	79.2
Widowed/Divorced	64	13.7
Unknown	28	6.0
Partnership		
No	43	9.2
Yes	404	86.7
Unknown	19	4.1
Employment		
No	196	42.1
Yes	252	54.1
Unknown	18	3.9
Education		
Compulsory schooling	53	11.4
Vocational Training	224	48.1
Upper secondary/University	146	31.3
Unknown	43	9.2
Number of children		
Two or less	214	45.9
More than two	212	45.5
Unknown	40	8.6
Household Income		
Up to and including 6000 CHF	325	69.7
More than 6000 CHF	105	22.5
Unknown	36	7.7

Abbreviations: Unknown, values are missing; n, number

## Results

### Study populations

Of the 575 contacted families, we received a response from at least one parent of 308 families (53.6% response rate, Table 1; Fig. 1; responders did not significantly differ from non-responding parents in socio-demographic and cancer-related characteristics as published in Baenziger et al. [20]. Of 478 responding parents, 12 did not answer the questions regarding LTFU. This study includes 466 parents –190 fathers (40.8%) and 276 mothers (59.2%)– of 302 long-term CCS (55.3% male). Parents’ mean age was 62.3 years (standard deviation (SD): 6.9 years, range 45–85, Table 1). Mean time since CCS’ diagnosis was 24.9 years (SD: 7.1, 7.8–40.9 years; Table 2). CCS’ average age at study was 32.3 years (SD: 6.4, range: 21–54). Of the 466 parents, 157 (33.7%, 98 mothers and 59 fathers) reported that their child attended LTFU (attenders), while 309 (66.3%, 178 mothers and 131 fathers) stated that their child does not attend LTFU or that they are unaware of it (non-attenders).

**Table 2:** Cancer-related characteristics of long-term childhood cancer survivors

Childhood Cancer Survivors (*N* = 302)		
	*n*	%
Gender		
Female	135	44.7
Male	167	55.3
Diagnosis (ICCC-3)		
Leukaemia	102	33.8
Lymphoma	55	18.2
CNS tumour	37	12.3
Neuroblastoma	13	4.3
Retinoblastoma	9	3.0
Renal tumour	20	6.6
Hepatic tumour	6	2.0
Bone tumour	14	4.6
Soft tissue sarcoma	22	7.3
Germ cell tumour	9	3.0
Langerhans cell histiocytosis	15	5.0
Treatment		
Surgery	35	11.6
Chemotherapy	166	55.0
Radiotherapy	82	27.2
Stem cell transplantation	19	6.3
Late effects †		
No	179	59.3
Yes	112	37.1
Unknown	11	3.6
	**Mean (SD)**	**Range**
Age at diagnosis, years	6.9(4.5)	0–15
Age, years	32.3(6.4)	21–54
Time since diagnosis, years	24.9(7.1)	7.8–40.9

Abbreviations: ICCC-3; International Classification of Childhood Cancer – Third edition; CNS, Central Nervous System; n, number; SD, standard deviation; † Parent-reported

### Aim 1: Parental involvement in LTFU care

**Fig. 3:**
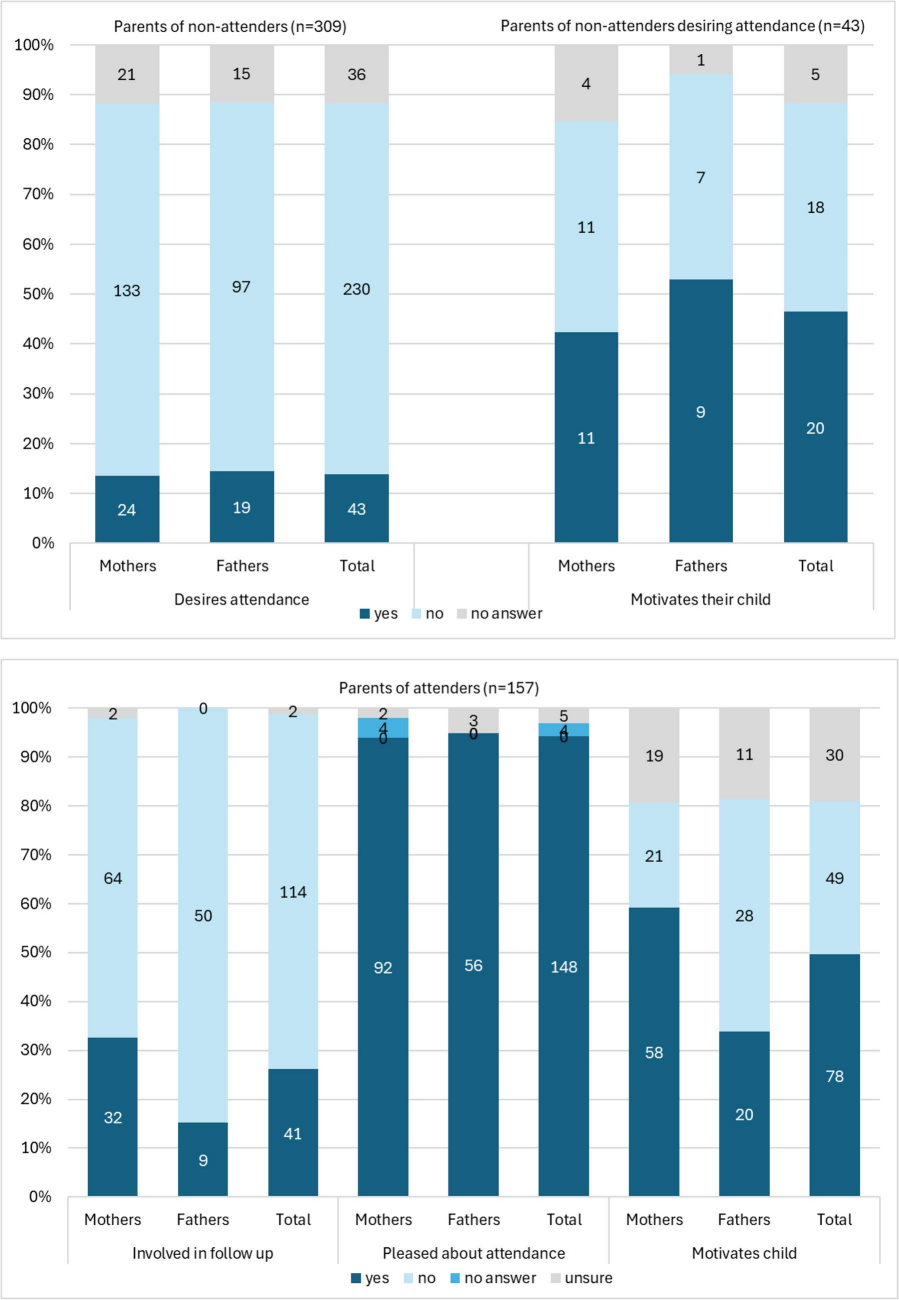
Parents’ involvement, attitude, and motivation regarding long-term follow-up care of adult childhood cancer survivors

Among parents of attenders, 114 (73.9%) stated *not* being involved in their child’s LTFU, while 41 (26.1%) remained involved. More mothers were involved than fathers (*n* = 32 (33.3%) vs. *n* = 9 (15.3%); χ^2^ (1,*N* = 155) = 6.14, *p* = 0.013, Fig. 3). In the open-ended questions, parents reported that their involvement in follow-up attendance entailed medical tasks, such as active communication with the physician, checking results and monitoring medication. They also described preventative and practical contributions, including providing survivors with information and reminding them about upcoming appointments). Finally, parents emphasized their emotional role, supporting their child through open communication, offering a sense of security, and discussing impact of results (Table 3).

**Table 3:** Themes of involvement, attitude, and motivation for long-term follow-up care of parents of childhood cancer survivors and corresponding quotes

	Medical	Prevention and practical	Emotional	Autonomy-related		Relationship
AIM 1. PARENTAL INVOLVEMENT						
Are you currently involved in your child’s follow-up? If yes, in which form?	*I'm checking that she's taking her medicines as well as possible and email contact with the endocrinologist* Mother of 19-year survivor	*Accompany to all doctor's appointments and provide linguistic support as well as organise transport services* Mother of 39-year survivor	*We talk about results of the doctor's visit* Father of 41-year survivor	n.a		n.a
AIM 2. PARENTAL ATTITUDES						
*I would like my child to attend LTFU, because:*	*Because there are still heart problems, orthopedic problems that are not managed by the treating medical doctor that treats my daughter* Mother of 18-year survivor	*To detect a possible relapse as soon as possible* Mother of 26-year survivor	*There is always some fear (uncertainty)* Father of 31-year survivor	*He is an adult and has to decide for himself* Mother of 12-year survivor		*Since it has been so long and the doctors know her* Father of 19-year survivor
*I would NOT like my child to attend LTFU, because:*	*Our son is cured* Father of 22-year survivor	*Our daughter sees her family doctor regularly and is well cared for. He has two “holistic” eyes on her health* Mother of 22-year survivor	*At one point, you have to be finished, in order to get some distance* Mother of 21-year survivor	*That is his decision, and I respect it* Father of 13-year survivor		n.a
AIM 3. REASONS FOR MOTIVATING OR NOT						
*I motivate my child to attend LTFU, by means of:*	*I ask and tell him how important the follow-up is* Mother of 30-year survivor	*I remind her of the annual check-ups she is required to perform but she willingly does them* Mother of 14-year survivor	*I support her wish for follow-up because it gives her (momentary) security and it is a prevention for her* Mother of 22-year old survivor		*I remind her of the annual check-ups, but she does them voluntarily* Mother of 16-year survivor	*We openly talk about it* Mother of 23-year survivor
*I do NOT motivate my child to attend LTFU, because of:*	*Disease dates back approx. 30 years, therapy fully completed* Father of 29-year survivor	*Is not necessary, for her it is absolutely clear to do this every year* Mother of 14-year survivor	*Because it would represent an emotional burden to the child* Father of 23-year survivor		*Because he is an adult and can decide for himself whether he wants to go for a follow-up check!* Mother of 22-year survivor	n.a
AIM 4. HEALTHCARE PROFESSIONALS INVOLVED; *multiple mentions possible*						
Specialities	**Most common** General/family practitioners (64.3%, *n* = 101) Adult oncologists (31.9%, *n* = 50)		**Common subspecialists** Endocrinologist (20.4%, *n* = 32) Ophthalmologist (19.7%, *n* = 31) Gynecologist (16.6%, *n* = 26) Pediatric oncologist (13.4%, *n* = 21) Neurologist (10.8%, *n* = 17) Nephrologist (5.1%, *n* = 8) Psychologist or psychotherapist (4.5%, *n* = 7)		**Further specialists** Dermatologist (*n* = 5) Cardiologist (*n* = 4) Gastroenterologist (*n* = 3) Dietician (*n* = 3) Otorhinolaryngology (*n* = 3) At previously treating hospital (*n* = 3) Orthopedist (*n* = 2) Otologist (*n* = 2) Pain management (*n* = 1) Andrologist (*n* = 1) Urologist (*n* = 1) Dentist (*n* = 1) Previous surgeon (*n* = 1) Nurse (*n* = 1)	
Ending LTFU	**Decision Maker** Treating physician or hospital (53.4%, *n* = 165 of 309) Childhood cancer survivor (18.4%, *n* = 57) Parents 5.2% (*n* = 16) Participative decision: healthcare professionals, parents, and sometimes including the CCS (17.5%, *n* = 54)		**Reasons for ending LFTU** Lack of prompts Long time since childhood cancer Insurance School conflicts			

### Aim 2: Parental attitude toward LTFU attendance

Among parents of attenders, 148 (94.3%) were pleased that their child attended LTFU, five unsure (3.2%), four not answering (2.5%), and none endorsing ‘no’. Proportions (yes vs. unsure) were similar among mothers: *n* = 92 vs. 2 and fathers: *n* = 56 vs. 3 (χ^2^ not tested given the small subgroups; Figure 3).

Reasons for desiring LTFU attendance included medical, prevention and practical, emotional, autonomy-related, and relationship reasons (Table 3). Parents most often referred to medical reasons, such as preventing relapse, monitoring for late effects or secondary malignancies, and managing side effects to maintain good health. Prevention and practical considerations were also emphasized, with several parents reporting that attendance helped placate fears of relapse or new health complications, especially as the cancer diagnosis dated back many years. Emotional reasons were equally important. Parents described a *“sense of security that all is going well*” when their child attended LTFU, and some highlighted that their child had felt abandoned after treatment, which reinforced the importance of continuing follow-up. Autonomy-related considerations also emerged, with some parents explaining that they personally did not see follow-up as necessary but nevertheless respected their child’s autonomous decision to attend. Finally, relationship factors influenced parental support. Some valued that the specialist already knew their child’s medical history, which made follow-up visits particularly meaningful.

Among parents of non-attenders, 230 (74.4%) parents did not desire their child to attend LTFU, 43 (13.9%) parents desired CCS to attend, and 36 (11.7%) did not answer. Proportions desiring attendance (yes vs. no) were similar among mothers (*n* = 24 vs. *n* = 133) and fathers (*n* = 19 vs. *n* = 97, χ2(2, *N* = 309) = 0.07, *p* = 0.996, Fig. 3). Explanations for not desiring their child to attend LTFU also followed the same reasoning (Table 3). From a medical perspective, many parents felt that routine medical visits were sufficient, or that their child was “*completely cured*”, often citing long-term remission, a physician’s reassurance, or the formal end of LTFU. Some even believed that their child’s health risks were no different from peers without a cancer history. Some specific medical characteristics – such as benign tumours, no symptoms, and feeling healthy – were also mentioned as reasons for not wishing to continue LTFU care. Practical arguments included reliance on medical checks provided by an employer, which were seen as an adequate substitute. Emotional motives centred on the wish to move on. Parents spoke of the need to forget the difficult period of illness, maintain a positive mindset, and avoid instilling fear through continued hospital visits. While some parents feared bad news, others were confident about their child’s health and felt attendance was unnecessary. Finally, autonomy-related reasons were again important, with parents stressing that survivors were “*old enough*” to make their own decisions — whether that meant continuing or discontinuing LTFU —depending on their age or cognitive maturity.

### Aim 3: Motivating their adult child’s LTFU attendance

Among parents of attenders, 78 (49.7%) stated to motivate their adult child to attend LTFU, while 49 (30.2%) stated *not* to motivate their child. Mothers of attenders were more likely to motivate their children than fathers (59.2%, *n* = 58 vs. 33.9%, *n* = 20, χ^2^(1,*N* = 127) = 11.40, *p* = 0.002, Fig. 2). Among the 43 parents of non-attenders who desired CCS to attend 46.5% (*n* = 20; 9 mothers, 11 fathers, χ^2^(1,*N* = 38) = 0.45, *p* = 0.504]) reported trying to motivate their child to attend LTFU.

Overall, both parents who motivated or chose *not* to motivate their children referred to medical, prevention and practical, emotional, autonomy-related, and relational aspects (Table 3). When parents described ways of motivating their child – for both attenders and non-attenders of LTFU care –, some highlighted medical reasons, such as a family history of unexpected childhood cancer, which underscored for them the importance of monitoring health and attending LTFU. Others focused on prevention and practical support, such as helping with scheduling appointments or even contacting physicians directly. Emotional motives were also present, with parents encouraging attendance because it offered both, them and their child, a sense of security. Some parents emphasized autonomy-related support, noting that their children were already independent in arranging their own follow-up, while others described relational strategies, such as engaging their child in conversations about the importance of LTFU. Conversely, parents who reported not motivating their child most often referred to medical arguments, stating that many years had passed since diagnosis or cure – sometimes 10 to 30 years – and thus they did not perceive LTFU as necessary. Practical reasons were also cited, with some parents simply noting “*no need*” for attendance. In terms of emotional reasons, several explained that illness remained a “*taboo topic*” in their family or feared that reminders might emotionally distress their child. Finally, autonomy-related perspectives were central. Many stressed their child’s adulthood, independence, and right to decide whether to attend, and accepted their child’s choice even if this meant foregoing LTFU.

### Aim 4. Healthcare professionals involved in LTFU and who decided to end LTFU

Parents of attenders reported a variety of healthcare professionals involved in LTFU, including general/family practitioners (64.3%, *n* = 101), adulthood oncologists (31.9%, *n* = 50), and/or subspecialties, including psychologists and psychotherapy (*n* = 7, 4.5%) (Table 3). This highlights that follow-up care is often provided across different professional groups. Parents of non-attenders reported that the decision to end LTFU care had been taken by the treating physician or hospital (53.4%, *n* = 165) or their child 18.4% (*n* = 57). Only 5.2% (*n* = 16) reported they (as parents) had decided, and 17.5% (*n* = 54) mentioned a participative decision, between the healthcare professionals, parents, and sometimes the CCS. In other words, the decision to end LTFU was most often initiated by healthcare professionals, but in some cases survivors themselves or shared decision-making processes played a role. Explanations for ending LTFU included systemic factors at the macro level, such as insurance coverage issues, the mesosystem, such as the lack of invitations by the clinics for LTFU and school conflicts, as well as personal factors at the micro level such as the long time since the cancer experience and survivors’ age.

## Discussion

This study described parents’ perspectives regarding LTFU of long-term CCS. Only few parents of adult CCS remained involved in their adult child’s LTFU. Mothers were more often involved and motivating their child than fathers. While most parents of attenders were pleased with their child’s LTFU attendance, only a quarter of parents of non-attenders wished that their child attended LTFU.

Our findings echo previous studies with parents reporting feeling ‘abandoned’ by the healthcare system shortly after treatment end, [21] and struggling to identify where their child could obtain LTFU [22]. Parents may not be fully aware of different LTFU models available since LTFU is often not standardised nor systematically implemented [23–27]. Parents can pass on this knowledge to CCS only if they understand the local system in place. A standardized transition approach from paediatric care to LTFU may help optimize the successful transfer [21]. A survivorship passport, as highlighted in studies from the US, Australia, New Zealand, and the EU, [28–30] containing information on the cancer history, medical treatments, and recommendations for LTFU [31] may represent a useful tool to guide parents and empower CCS with growing independence and self-management. Ideally, open communication between parent and child should be supported. However, as CCS grow older, they might feel uncomfortable sharing health-related or psychological experiences with their parents. This highlights the need for a nuanced understanding of parental involvement: support may include both active encouragement and respectful withdrawal as survivors take ownership of their follow-up care [32]. North American studies show that parental involvement adapted to the young persons’ developmental stage was found to support successful transition to adult care, whereas overbearing or “helicopter” parenting tended to impede this process [33].

Parents accepted their child's decision about LTFU attendance, supporting individual choice and independence. Our findings highlight that understanding relational autonomy is important: autonomy is the capability to make independent, self-governed decisions; relational refers to the influence of the environment, including relationships and social context on their decision-making process [34]. Social and ‘relational’ factors, such as parental or healthcare professionals’ support, motivation, and engagement, can significantly impact a CCS’s decision to participate in LTFU [4, 7, 34]. Parents’ narratives surrounding treatment and care might influence CCS’s attitudes towards the healthcare system, such as the belief to benefit and being able to adhere to recommended LTFU [35]. CCS have mentioned attending LTFU as a way to show appreciation to healthcare professionals, feeling they owed it to them due to the excellent care received [36]. By conceiving autonomy as a relational trait, we might better understand and address the social context in CSS's healthcare decisions to ultimately enhance LTFU attendance and improving their long-term outcomes. LTFU programs could therefore explicitly value relational factors—such as family involvement, peer support, and patient–provider relationships—as resources that support, rather than constrain, autonomous decision-making. In practice, this means healthcare systems could promote autonomy by fostering trusting long-term relationships, actively involving parents in transition processes, and ensuring continuity of care teams to provide sustained support for decision-making.

Parental involvement in LTFU offers a significant advantage as they have a comprehensive knowledge of CCS’ health since childhood. Parents have stated a need for additional information, for managing late effects, and the future [3, 22, 37]. With more time after diagnosis and decreasing LTFU involvement, this gap might exacerbate [11]. Continuous parent/patient education is, therefore, vital [11]. When developing education materials for parents and CCS, it is important to consider that mothers’ and fathers’ involvement in LTFU differ, with mothers more actively involved than fathers. This aligns with the traditional caregiving roles still predominant in Switzerland [38]. An increase in traditional role distribution has been observed in response to managing childhood cancer, where mothers spending most of their time caring for the child, and fathers working more to ensure financial stability despite medical expenses [39–42]. Since fathers have been less involved in caretaking, their help could be valuable in fostering a social norm regarding LTFU attendance.

### Study limitations

Our conclusions are limited regarding the directionality of associations because we used a cross-sectional design, highlighting the need for longitudinal studies to track evolving engagement in survivors’ long-term follow-up. Self-selection bias might also affect our findings as parents who are more involved or concerned about their children’s health may be more likely to participate in a questionnaire on the health and well-being. We did not have any information whether the CCS’ experienced medical late effects. However, as a proxy, we included registry data on relapse and self-reported data on late effects. Additionally, only parents’ perspectives were assessed, while including adult survivors’ views could have provided a more complete picture of parental involvement. A further limitation is the dichotomic categorization of attender’s vs non-attenders: parents’ lack of awareness of survivors’ LTFU may not necessarily reflect the survivor’s actual LTFU (non-)attendance. There is also a potential for social desirability bias in parents’ self-reports; however, the generally low levels of reported involvement and candid open-ended responses (e.g., ‘my child decides him/herself’) suggest that parents answered honestly.

The major strengths of our study include that we used a population-based sample of parents with an exceptionally high number of participating fathers, who are often underrepresented in research. We are also one of the first research groups to examine involvement, attitude, and motivation for LTFU among parents of very long-term CCS.

### Clinical implications

Parents trust their children’s decision on attending LTFU but continue their support through motivation. Although parents' involvement may be constrained by confidentiality, and the legal age of long-term CCS requiring consent, healthcare professionals can play a crucial role in fostering a social norm during survivorship. They can achieve this through communication with parents and CCS, such as guiding them in navigating the LTFU models in their region. Designing LTFU systems to facilitate appropriate engagement of parents and CCS is essential. A study reported that many CCS felt LTFU was poorly coordinated in a fragmented system, which led to dissatisfaction [43]. Practical aspects such as assistance with scheduling and navigating the healthcare system may be auspicious (e.g., messages to prompt attendance). Providing adequate information at key timepoints of the survivorship period is necessary. To support both survivor autonomy and appropriate parental engagement, healthcare providers—including GPs, who are the most common LTFU providers—may benefit from training and resources on transition communication and shared decision-making.

Moreover, despite the prominence of psychological factors in qualitative findings, mental health professionals were rarely mentioned in reports of LTFU care (psychologist or psychotherapist: 4.5%, *n* = 7), suggesting a gap between identified needs and documented care roles. Digital portals coordinated with involved healthcare professionals where CCS can view information and ask questions could enhance access to information, increase the visibility of mental health services available, improve communication, and promote low-threshold support [36].

## Conclusion

Parents still play an important role in the LTFU of adult CCS, despite no longer being directly involved. There is untapped social and environmental potential to increase CCS’ LTFU attendance. The variety of LTFU models can be difficult to navigate; thus, working to improve visibility and encouragement might help increase attendance.

## Data Availability

The datasets generated and/or analysed during the current study are not publicly available due to confidentiality but are available in de-identified form from the corresponding author on reasonable request.

## Abbreviations

CCS: Childhood Cancer Survivors
CHF: Swiss francs
ICCC-3: International Classification of Childhood Cancer – Third Edition
LTFU: Long term follow-up
SCCR: Swiss Childhood Cancer Registry Switzerland
SCCSS-Parents: Swiss Childhood Cancer Survivor Study – Parents
